# Supplementary figures and images for: In vivo multiplexed modeling reveals diverse roles of the TBX2 subfamily and Egr1 in Kras-driven lung adenocarcinoma
Source: Genes Dis. 2025 Sep 3;13(3):101840. doi: 10.1016/j.gendis.2025.101840 (PMC12907852; doi:10.1016/j.gendis.2025.101840)

# Supp-1

**A**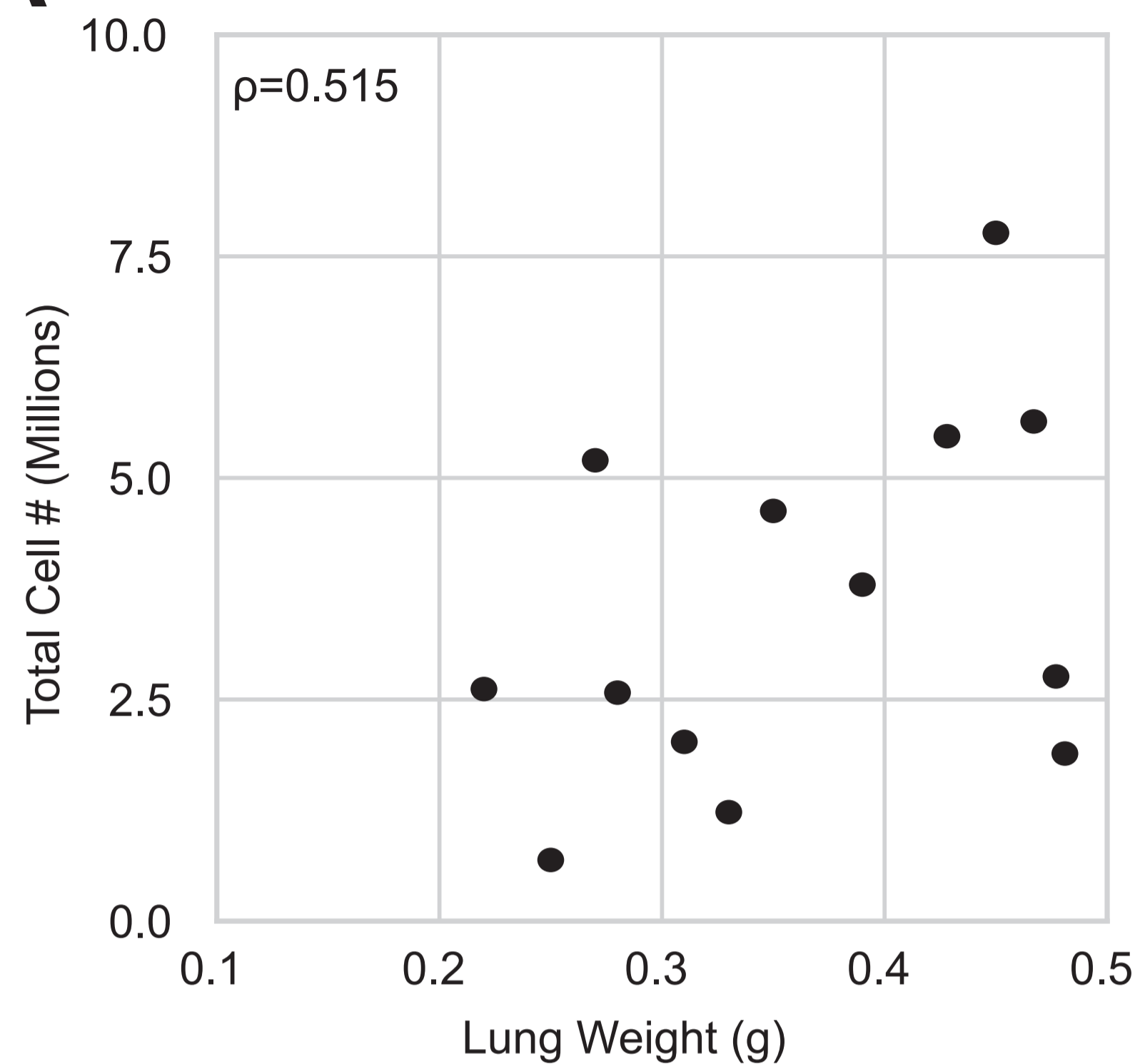**B**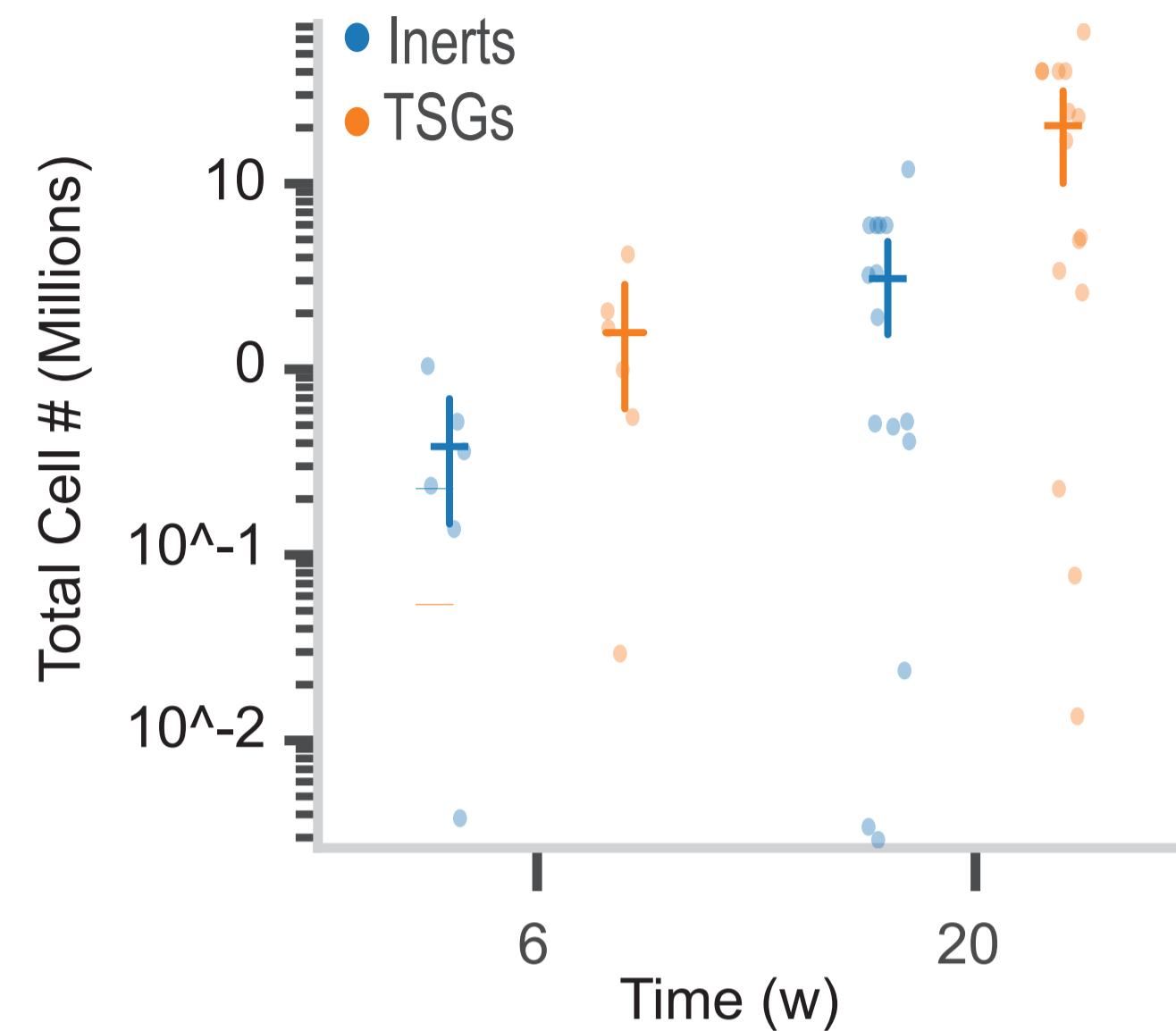**C**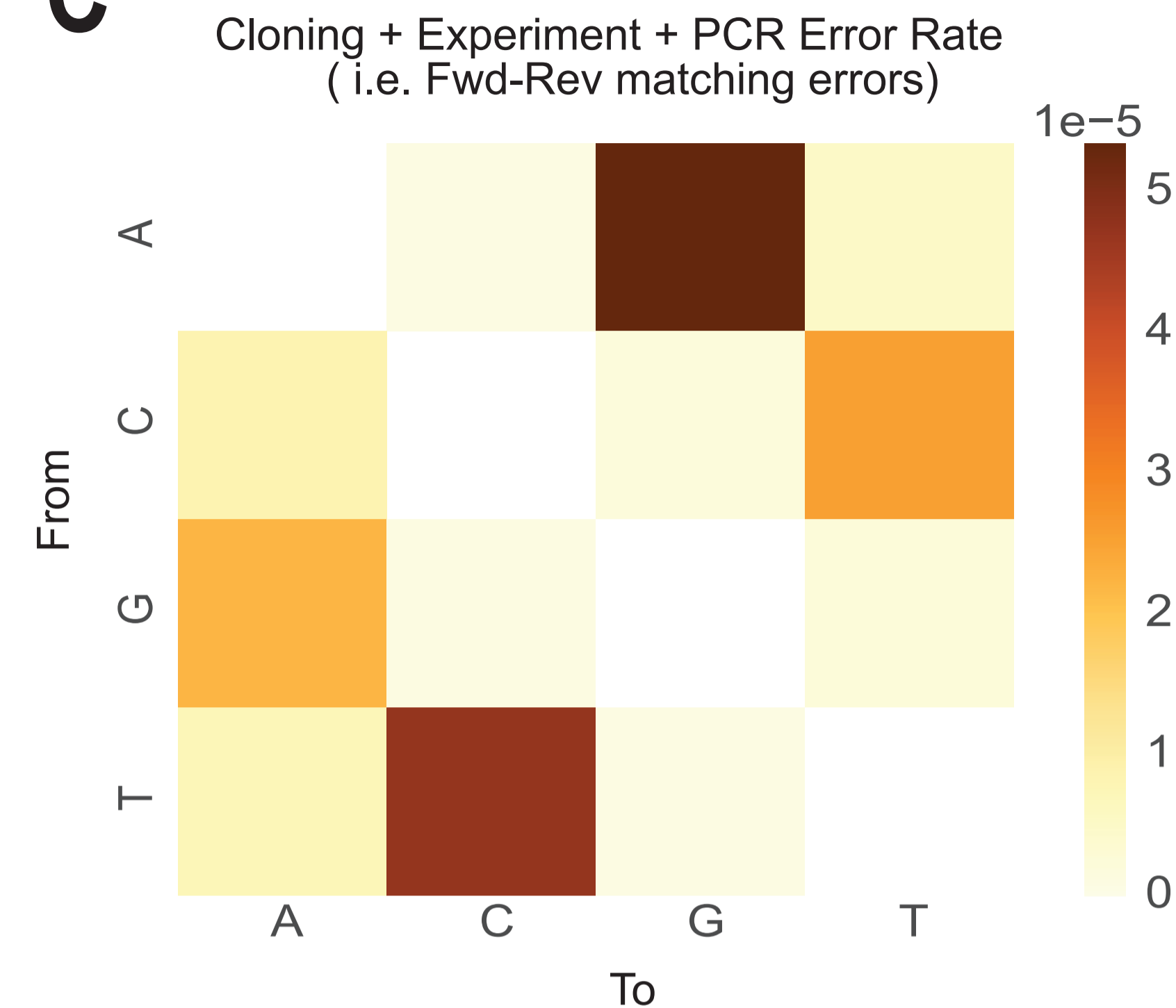**D**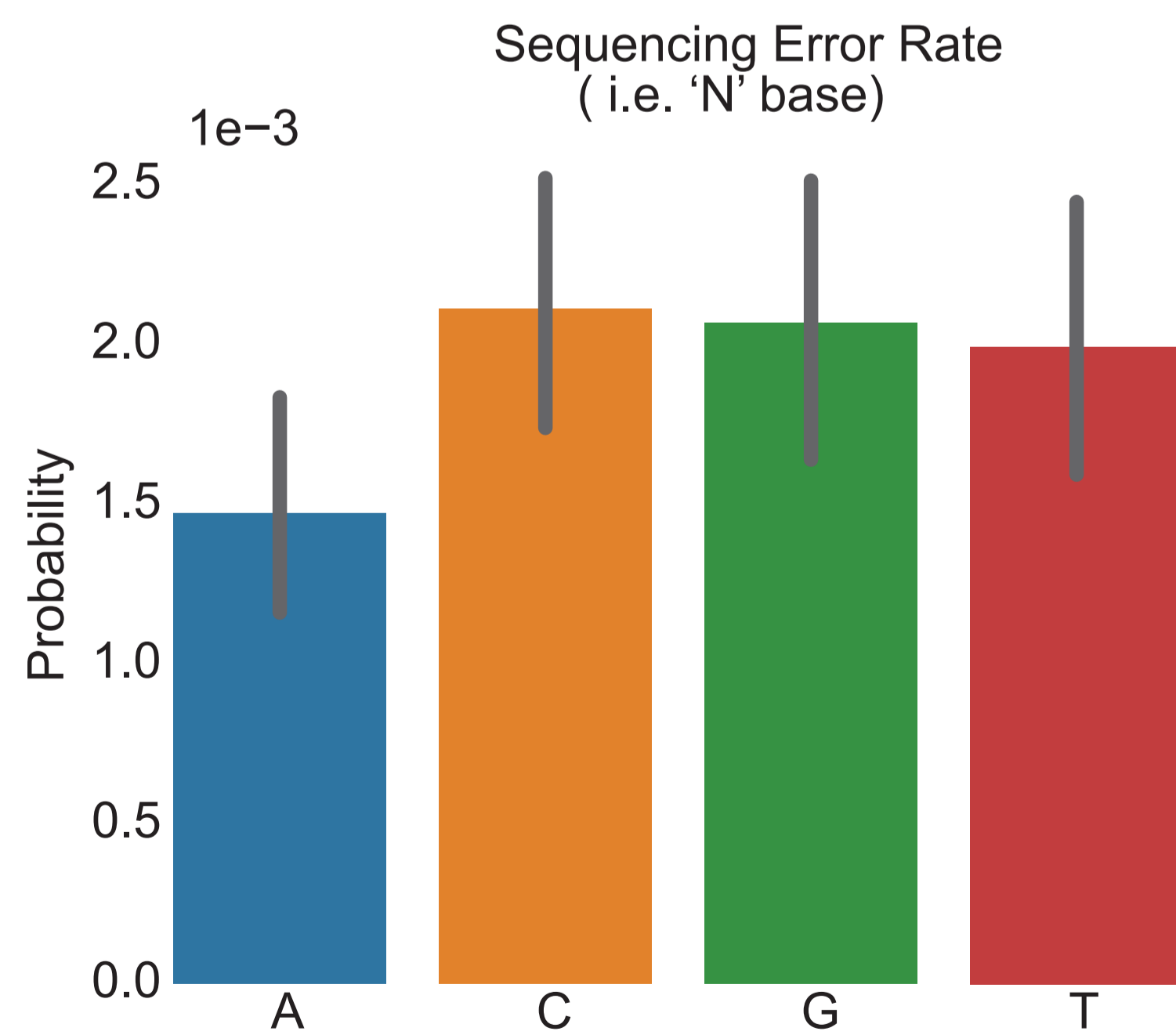**E**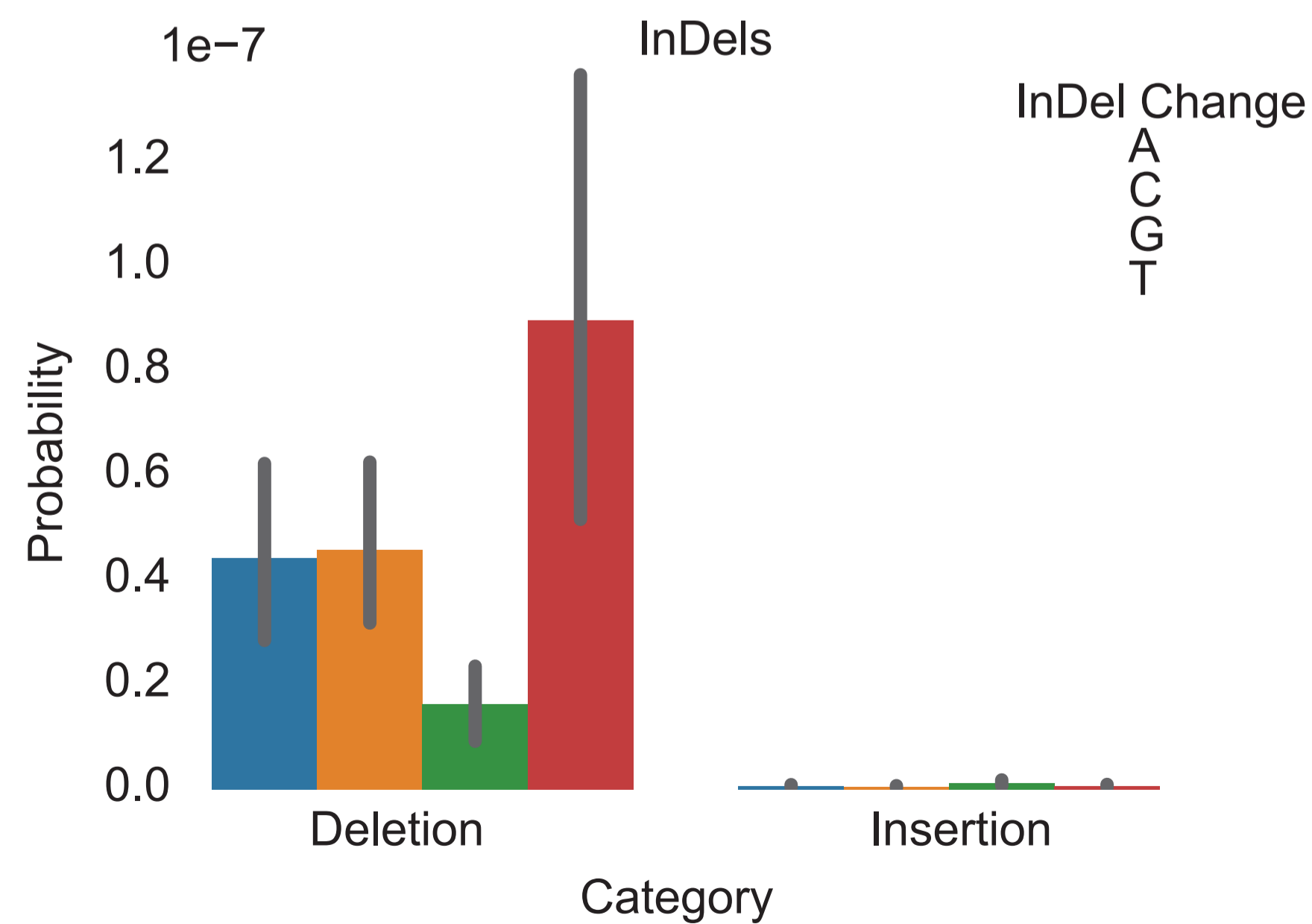**F**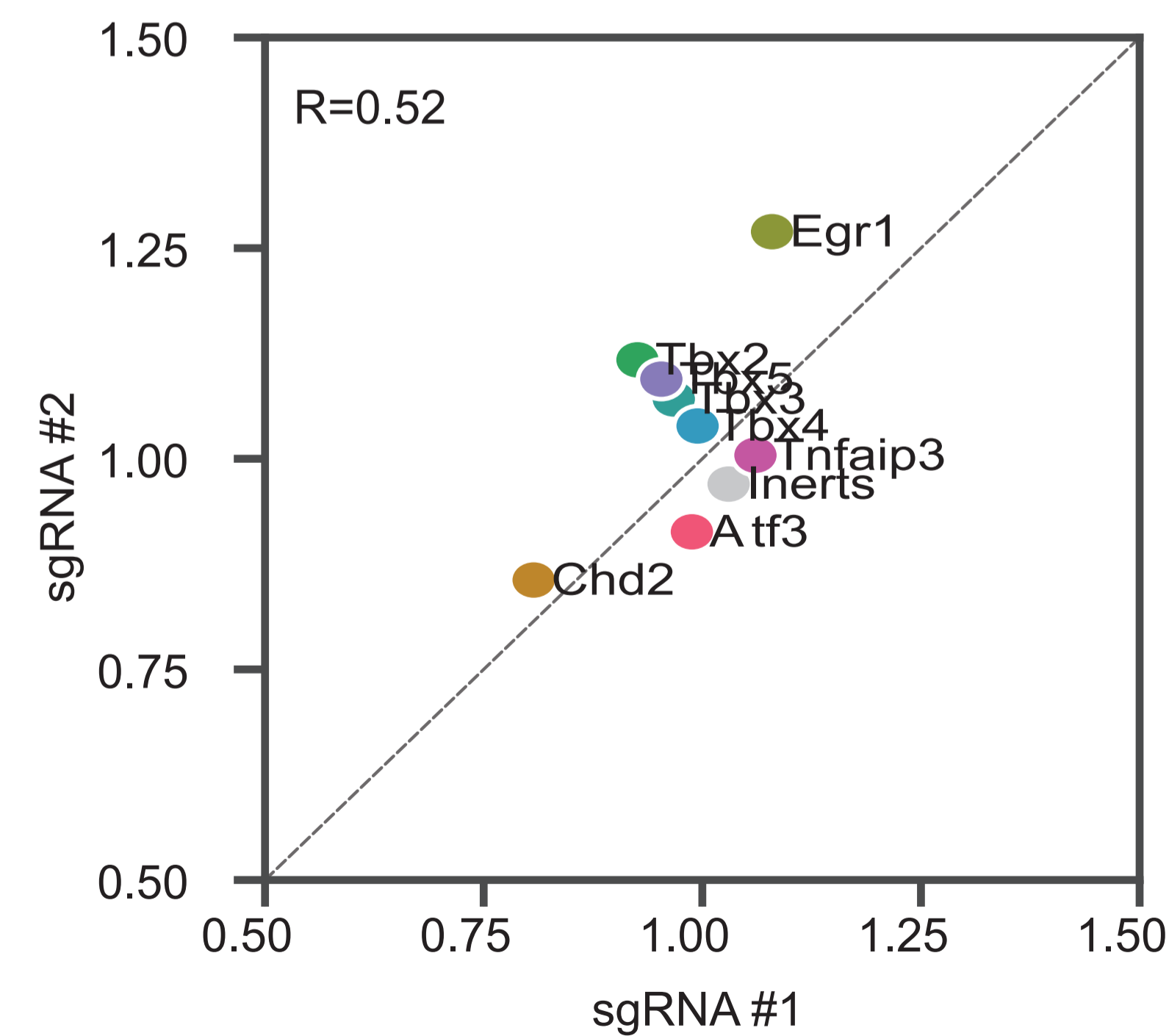

Supplement: Multimedia component 1 [file mmc1.pdf]

A

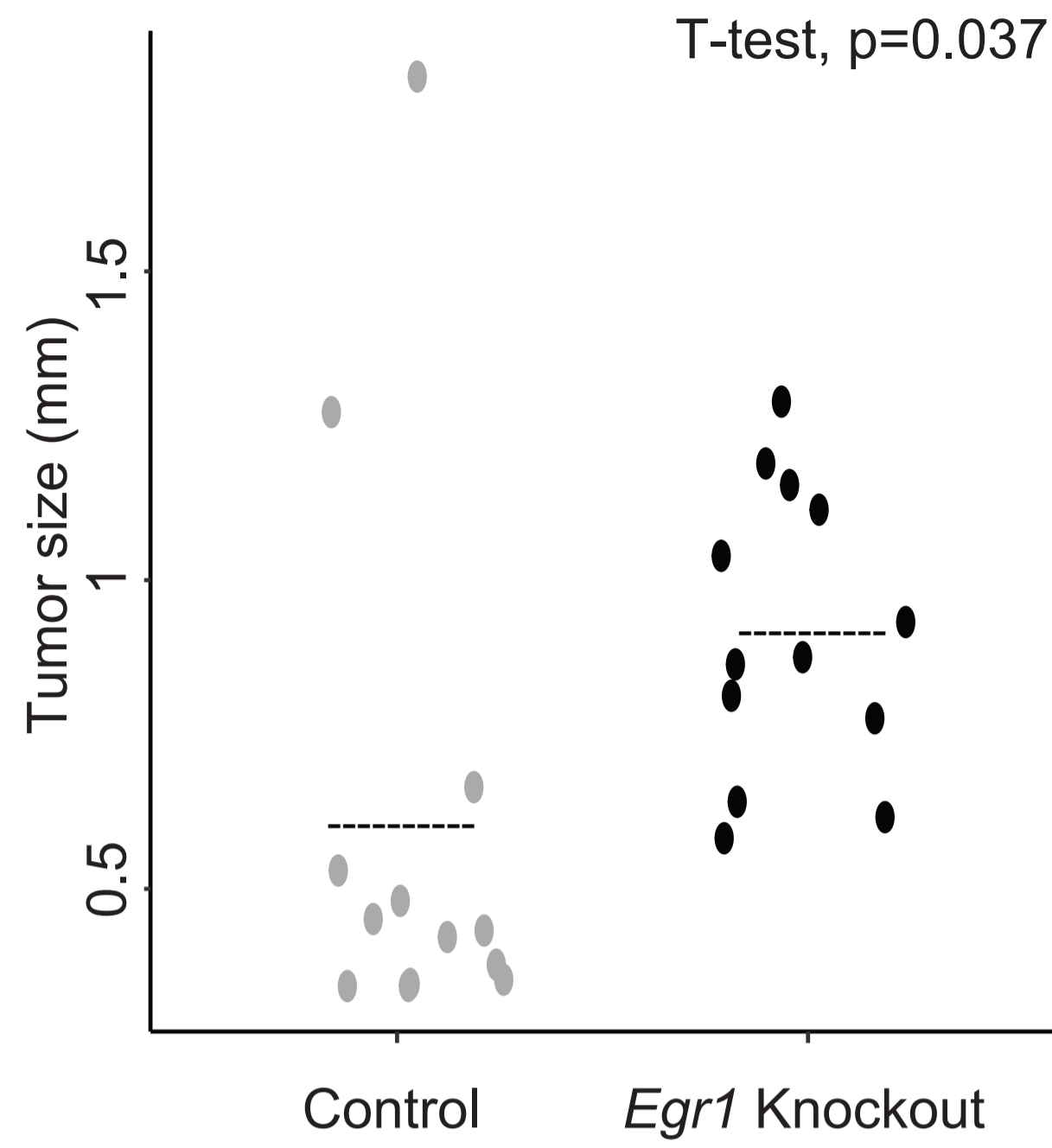

B

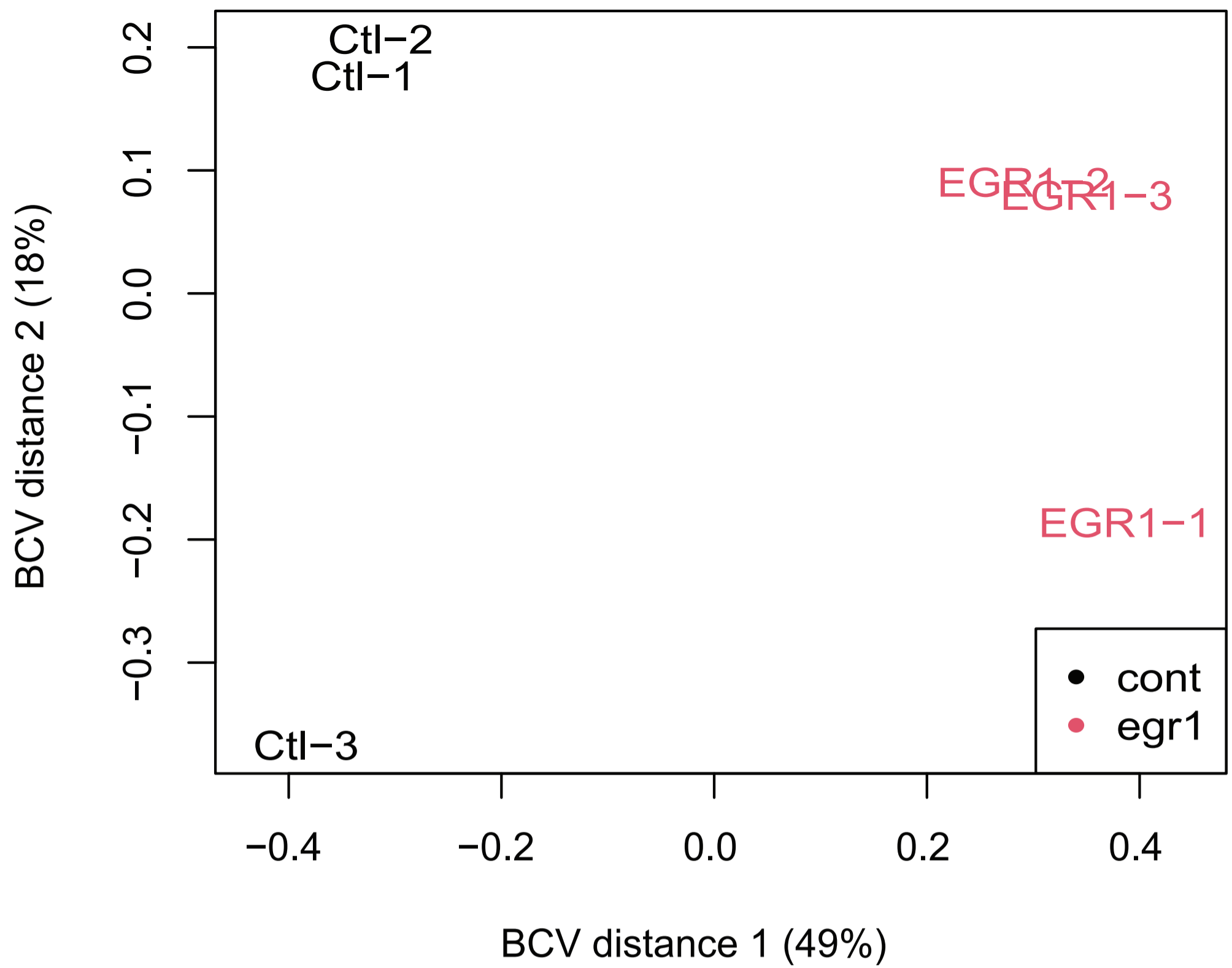

C

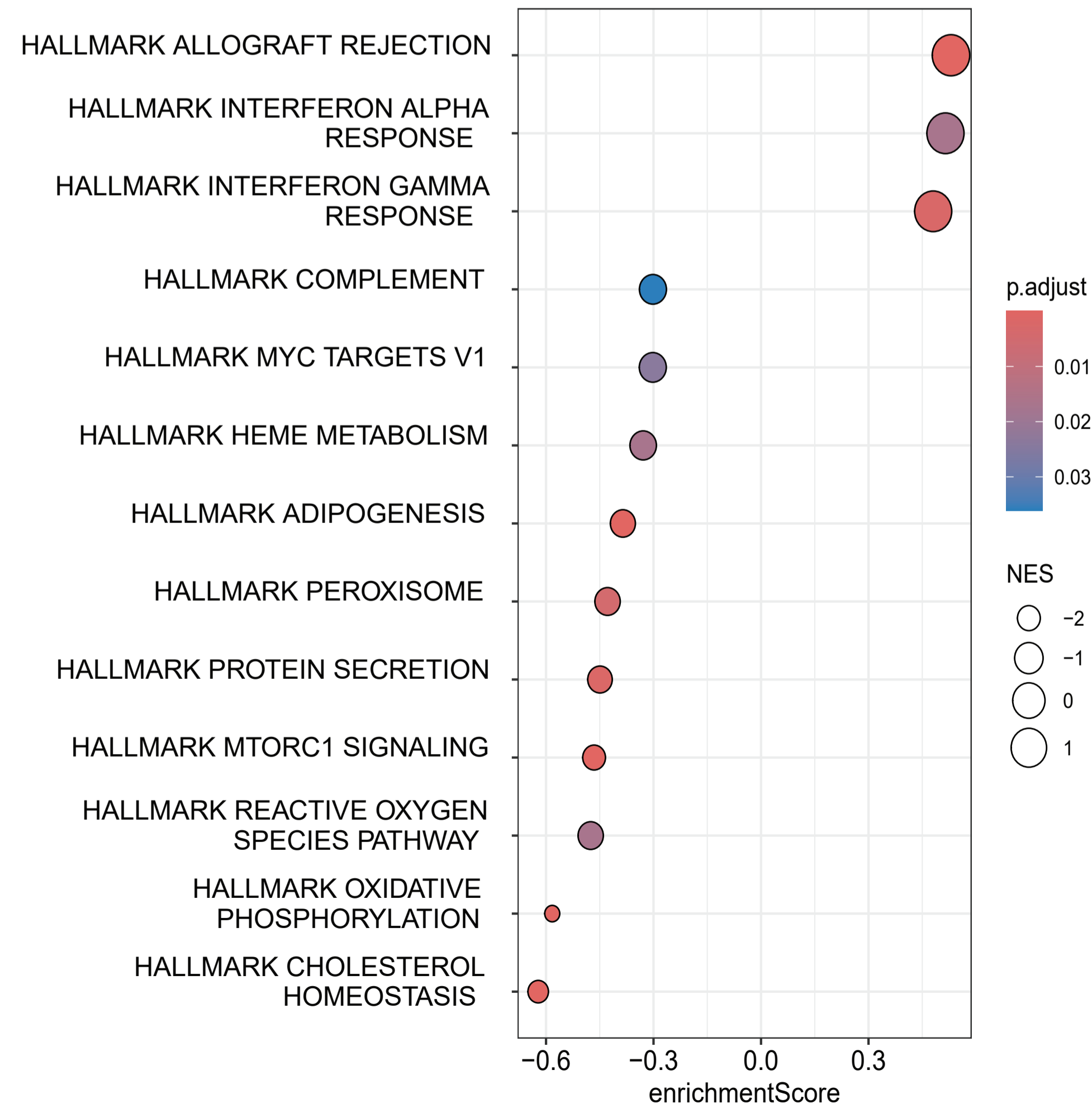

D

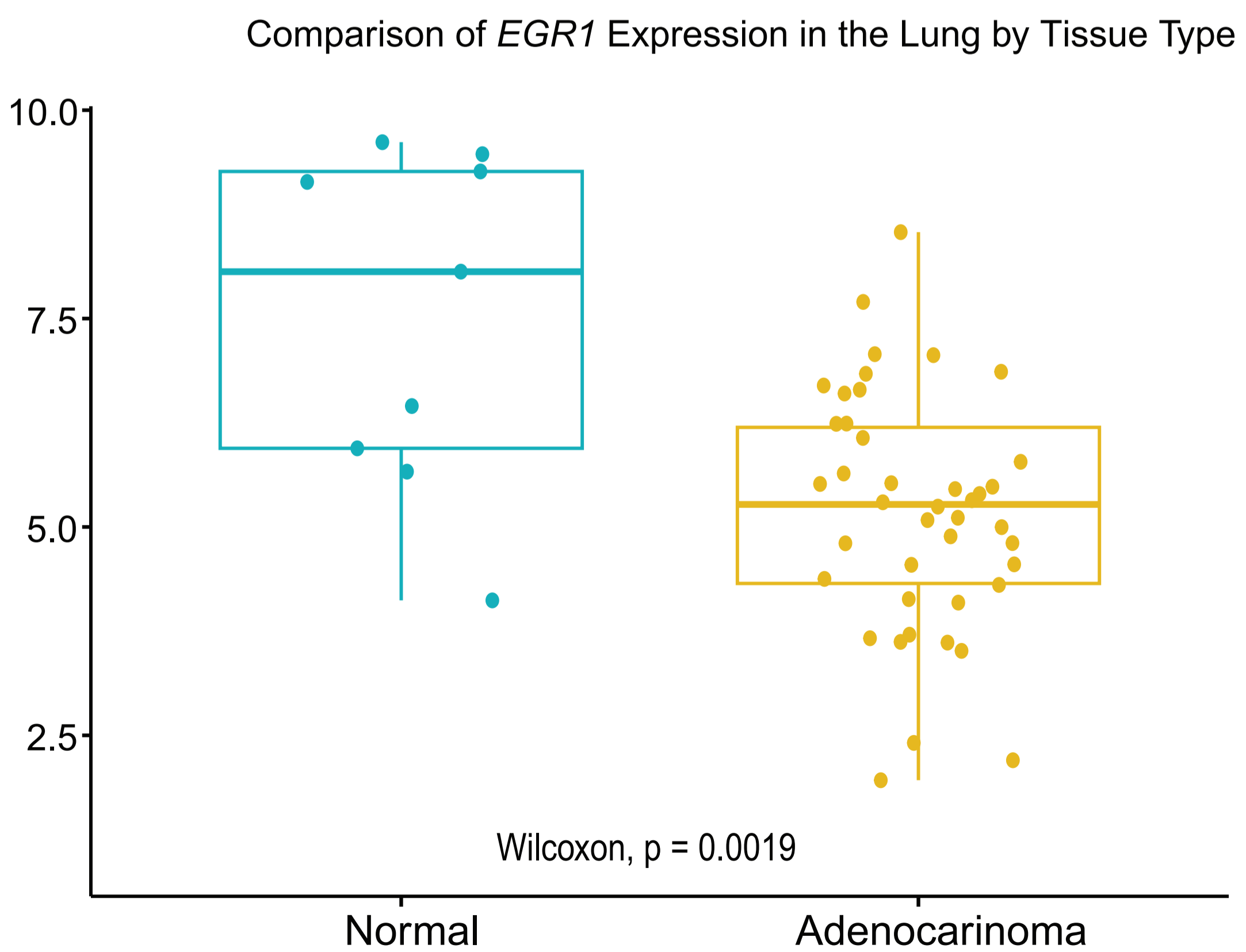

Supplement: Multimedia component 3 [file mmc3.pdf]
