# Supplementary material for: In vivo multiplexed modeling reveals diverse roles of the TBX2 subfamily and Egr1 in Kras-driven lung adenocarcinoma
Source: Genes Dis. 2025 Sep 3;13(3):101840. doi: 10.1016/j.gendis.2025.101840 (PMC12907852; doi:10.1016/j.gendis.2025.101840)

Supp-2

A

sgRNA	6 weeks Mean (relative to inerts)	6 weeks P-Value	20 weeks Mean (relative to inerts)	20 weeks P-Value
Atf3	0.885	0.164	0.948	0.274
Chd2	0.789	0.009	0.832	0
Egr1	1.340	0	1.129	0.004
Tbx2	0.707	0	0.999	0.983
Tbx3	0.933	0.403	1.026	0.602
Tbx4	1.038	0.662	1.024	0.603
Tbx5	0.903	0.235	1.019	0.710
Tnfaip3	1.188	0.028	1.035	0.472
Rb1	1.115	0.166	2.501	0
Pcna	0.863	0.098	0.940	0.198

B

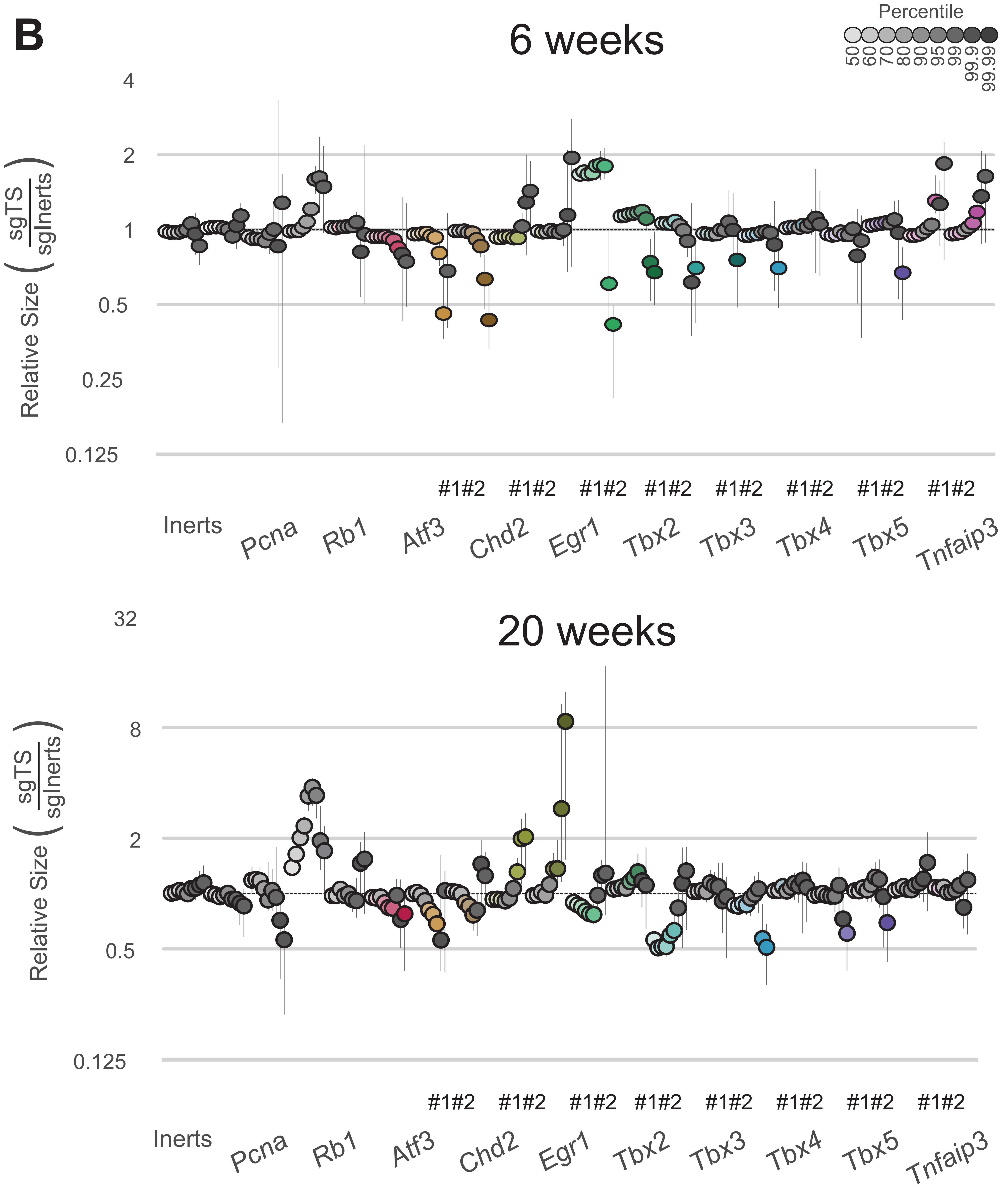

Supplement: Multimedia component 2 [file mmc2.pdf]
