# Supplementary material for: In vivo multiplexed modeling reveals diverse roles of the TBX2 subfamily and Egr1 in Kras-driven lung adenocarcinoma
Source: Genes Dis. 2025 Sep 3;13(3):101840. doi: 10.1016/j.gendis.2025.101840 (PMC12907852; doi:10.1016/j.gendis.2025.101840)
Supplement: Multimedia component 4 [file mmc4.pdf]

Supplementary Table- S1: Lenti-sg*RNA* vectors used for gene targeting. The table lists the sgRNA sequences used to target each of the genes analyzed in this study and their corresponding sgIDs. sgRNAs targeting *Rb1* (Addgene #89647), *Neo1* (Addgene #67594), *Neo3* (Addgene #89653), and *Pcna*, has been adopted from previous studies(16).

sgRNA target	sgRNA number	sgRNA Sequence	PAM Sequence	Exon Number	Target Cut Length	Target Cut %	On-Target Efficacy Score	sgID
Tbx2	#1	GCTCGCACGATGTGGAATCG	CGG	3	699	32.7	0.6592	TTGGCAAC
Tbx2	#2	GTCCGGCCACAGGGGAACAG	TGG	2	591	27.7	0.6211	AATGCGTG
Tbx3	#1	GAGCACCTCACTTTAAACGG	AGG	2	405	18.7	0.6961	TTTACCCG
Tbx3	#2	CATCATGGATCAGTTAGTGG	GGG	1	205	9.5	0.6828	TTGCCTGT
Tbx4	#1	CTTGTAGCGATGGTCATCTG	CGG	3	382	23	0.6082	TGAGCTTG
Tbx4	#2	CCCGGATTCTCCTGCCACCG	GGG	4	484	29.2	0.5905	TTGTCCGA
Tbx5	#1	TGGCTGAAGTTCCACGAAGT	GGG	3	206	13.2	0.6608	CAGTCGTA
Tbx5	#2	CGAAACCTGAGAGTGCTCTG	GGG	2	93	6	0.7005	ATCGTTGC
Egr1	#1	GAGGATTGGTCATGCTCACG	AGG	2	476	29.7	0.6483	CGATTAGG
Egr1	#2	GTTATCCCAGCCAAACGACT	CGG	2	366	22.8	0.6527	CTTACGGT
Tnfaip3	#1	ACTGACAAGCTGCATGCATG	AGG	3	314	13.5	0.6959	TTCCTCCT
Tnfaip3	#2	AACCATGCACCGATACACGC	TGG	2	139	6	0.5872	GCGGAATA
Chd2	#1	AAGCAACCTAAGATTCAGCG	TGG	7	593	10.8	0.684	TTCCAAGC
Chd2	#2	GTCTTATATTCACAGCACAT	AGG	4	315	5.7	0.4723	TCAGTTCG
Atf3	#1	TCAAATACCAGTGACCCAGG	AGG	2	78	14.3	0.7489	ACTTGGTC
Atf3	#2	GGCGGTCGCACTGACTTCTG	AGG	2	37	6.8	0.6238	TAGCCTGT
