# Supplementary material for: In vivo multiplexed modeling reveals diverse roles of the TBX2 subfamily and Egr1 in Kras-driven lung adenocarcinoma
Source: Genes Dis. 2025 Sep 3;13(3):101840. doi: 10.1016/j.gendis.2025.101840 (PMC12907852; doi:10.1016/j.gendis.2025.101840)
Supplement: Multimedia component 5 [file mmc5.pdf]

Supplementary Table- S2: Lung cancer cell lines harboring KRAS mutations adopted from OmicsSomaticMutations dataset

	KRAS Lung cancer Cell line name
1	NCI-H2887
2	Calu-6
3	NCI-H2122
4	HCC-461
5	NCI-H647
6	NCI-H1944
7	Lu-65
8	LU99
9	NCI-H460
10	NCI-H1792
11	Calu-1
12	NCI-H2030
13	LCLC-97TM1
14	NCI-H441
15	COR-L23
16	NCI-H1355
17	HCC-44
18	IA-LM
19	SW 1573
20	A549
21	NCI-H2291
22	A427
23	RERF-LC-Ad2
24	NCI-H727
25	SHP-77
26	RERF-LC-Ad1
27	NCI-H1373
28	MOR/CPR
29	NCI-H358
30	HOP-62
31	HCC515
32	NCI-H2009
33	NCI-H23
34	NCI-H1573
35	NCI-H157-DM
36	NCI-H650
37	NCI-H1155
38	A549_CRAF_KD
