# Supplementary material for: In vivo multiplexed modeling reveals diverse roles of the TBX2 subfamily and Egr1 in Kras-driven lung adenocarcinoma
Source: Genes Dis. 2025 Sep 3;13(3):101840. doi: 10.1016/j.gendis.2025.101840 (PMC12907852; doi:10.1016/j.gendis.2025.101840)
Supplement: Multimedia component 6 [file mmc6.pdf]

Supplementary Table- S3: Putative Stage-Specific Effects of Gene Knockouts in KRAS-Driven Lung Adenocarcinoma Based on *In Vivo* Tuba-seq Screening. ⬆️ / ⬇️ = Increase or decrease in tumor phenotype upon gene knockout. (*****) = p-value significance from the study

**Gene KO**	**Initiation**	**Growth**	**Progression**	**Summary Interpretation**
Egr1	⬆️ Strong ↑↑↑ (***)	⬆️ Moderate ↑ (***)	⬆️ Strong ↑↑ (**)	Strong tumor suppressor at all stages
Tnfaip3	⬆️ Strong ↑ (****)	⬆️ Slight ↑ (*)	No significant effect	Mild tumor suppressor; promotes progression
Tbx4	⬆️ Moderate ↑ (*)	No significant effect	No significant effect	Possibly redundant/inert
Tbx3	No significant effect	No significant effect	No significant effect	Possibly redundant/inert
Tbx5	No significant effect	No significant effect	No significant effect	Possibly redundant/inert
Atf3	⬆️ Moderate ↑ (***)	No significant effect	No significant effect	Mild early tumor suppressor
Chd2	⬆️ Moderate ↑ (*)	⬇️ Moderate ↓ (*)	No significant effect	Context-dependent; biphasic role
Tbx2	⬆️ Strong ↑↑↑ (****)	⬇️ Moderate ↓ (***)	⬆️ Moderate/strong↑ (*)	Context-dependent; biphasic role
